# Risk factors and outcomes after unplanned extubations on the ICU: a case-control study

**DOI:** 10.1186/cc9964

**Published:** 2011-01-13

**Authors:** Robin I de Groot, Olaf M Dekkers, Ingeborg HF Herold, Evert de Jonge, M Sesmu Arbous

**Affiliations:** 1 Department of Intensive Care, Leiden University Medical Center, Albinusdreef 2, Leiden, 2300 RC, The Netherlands; 2 Department of Clinical Epidemiology, Leiden University Medical Center, Albinusdreef 2, Leiden, 2300 RC, The Netherlands; 3 Department of Endocrinology and Metabolic Diseases, Leiden University Medical Center, Albinusdreef 2, Leiden, 2300 RC, The Netherlands; 4 Department of Intensive Care, Catharina Hospital Eindhoven, Michelangelolaan 2, Eindhoven, 5623 EJ, The Netherlands

## Abstract

**Introduction:**

Unplanned extubation (UE) is a frequent event during mechanical ventilation in critically ill patients and might be associated with increased morbidity and mortality. However, detailed knowledge of risk factors and outcomes after UE is lacking.

**Methods:**

A case-control study was performed with a case to control ratio of 1:4. Incidence density sampling was applied. Seventy-four cases and 296 control patients were included.

**Results:**

Seventy-four UEs occurred in 69 patients, comprising 2% of all mechanically ventilated patients. Multivariable regression analysis revealed that the first and second categories of the Ramsay Sedation Scale score were associated with a high risk for an UE (odds ratios (ORs) 30 and 25, respectively). Male sex, subunit of the intensive care unit (ICU), length of stay in the ICU and midazolam use at time of UE were also risk factors for an UE. Patients with an UE had lower hospital mortality than mechanically ventilated patients without UE, 10% versus 30%, respectively. Forty-seven percent (*n *= 35) of the patients with an UE had to be reintubated.

**Conclusions:**

The present study shows that the first and second categories of the Ramsay Sedation Scale were associated with a high risk for an UE. Also, male sex and use of midazolam at time of UE were identified as risk factors for an UE. However, compared with mechanically ventilated controls, no increased mortality was shown for UE patients. In UE patients without the need for subsequent reintubation, mortality was very low.

## Introduction

Unplanned extubation (UE) is a frequent event after endotracheal intubation for respiratory support in critically ill patients and is associated with increased morbidity and mortality [1-12]. The incidence of UE among intubated patients is reported to vary from 0.3% [7] to 14% [7,13], depending on patient characteristics, the characteristics of the intensive care units (ICUs) surveyed and the duration of mechanical ventilation of the patients [14]. UEs account for approximately 10% (range, 3% to 16%) of extubations and require reintubation in 60% of the cases [4]. Furthermore, experiencing an UE is associated with prolonged duration of mechanical ventilation, ICU stay and hospital stay compared to not having experienced an UE [4,15,16]. Reported risk factors for UE include route of tracheal intubation, method of tube fixation [3] and method and level of sedation [3,17].

Unplanned extubation is defined as a premature removal of the endotracheal tube by action of the mechanically ventilated patient (deliberate unplanned extubation) [17] or premature removal during nursing and medical care (accidental extubation) [18]. Although UE has been studied regularly, many questions about incidence, determinants and outcomes of UE have not been answered in all detail. Moreover, inconsistent findings exist, especially with respect to outcomes after UE, with some authors reporting improved outcomes after UE [14,15]. This may be explained by differences in study design, study population and ICU characteristics.

Understanding the determinants of UE is critical for risk assessment in individual patients and for developing interventions to reduce the incidence of this mechanical ventilation complication. We therefore aimed to study the incidence, determinants and outcomes of UE and to asses the risk factors for reintubation in full detail. The tertiary care ICU setting in which this case-control study was performed represents the full spectrum of clinical problems in contrast to other studies. Furthermore, we were able to obtain extensive clinical information for cases and controls from automated clinical data registers.

## Materials and methods

### Study design and definitions

This prospective case-control study was conducted in a tertiary care ICU. From 1 December 2005 to 1 June 2008, all patients requiring an artificial airway (orotracheal or nasotracheal tube) at one of the three subunits of the ICU of the Leiden University Medical Center (Leiden, the Netherlands) were monitored for the occurrence of an UE. Cases were consecutive patients with an UE within the study period. For the purpose of the study, UE was defined as premature removal of the endotracheal tube by action of the patient. Patients who experienced an accidental extubation during nursing and medical care were not included among the cases studied.

For the selection of controls, incidence density sampling was used, thereby matching the controls on time [19]. For every occasion of an UE, four control patients were randomly selected from among all mechanically ventilated ICU patients present at the time an UE occurred. Controls were not matched to cases with respect to clinical characteristics such as age and sex. The reason for not matching on such variables was twofold: First, after matching, the effect of the matched variables on the outcome cannot be assessed; second, matching can introduce bias in case-control designs.

This study conforms to the provision of the Declaration of Helsinki in 1975 (revised in 2008 in Seoul, South Korea) [20]. None of the patients was exposed to any intervention for study purposes. Given the observational nature of the study and the fact that collecting information on UE is standard practice for both the Safety Management Policy of our hospital and the quality practice of the Dutch Association of Intensivists, according to the local institutional review board the written, informed consent of the patient was not deemed necessary.

### Study setting and treatment procedures

Patients from the three adult tertiary care ICU subunits at the Leiden University Medical Center, with a capacity of 29 beds, were included. Each subunit facilitates the mixed ICU population, although some preference exists for surgical patients to be allocated to the two subunits in the vicinity of the operating rooms. The population represents a mixture of patients with complex medical conditions and patients undergoing planned and emergency surgical, thoracosurgical and neurosurgical procedures. The ICU is staffed by board-certified critical care specialists, trainees in critical care medicine and medical residents, who provide 24-hour in-unit coverage. Nursing staff work in three shifts: 7:30 AM to 3:00 PM, 3:00 PM to 10:30 PM and 10:30 PM to 7:30 AM. The patient-to-nursing staff ratio is 1½:1 during daytime, 1¾:1 in the evening, and 2:1 during nighttime.

The preferred route of intubation at our institution is oral. Tracheal tubes are routinely secured with cotton tape tied around the patient's head. Physical restraints are used when deemed necessary by the nursing staff. Either midazolam or propofol, alone or in combination with morphine, methadone or sufentanil, is used for sedation. In every patient, the ventilatory support and the level of sedation are adjusted to the specific clinical requirements. Furthermore, we systematically apply a weaning protocol on all participating ICU units. The weaning protocol states the following:

1. Criteria for start of the weaning process, including:

1.a. Reversal of initial critical illness.

1.b. Adequate oxygenation with respect to fraction of inspired oxygen (FiO_2_) <50%, oxygen partial pressure (pO_2_)/FiO_2 _ratio >20-26 kilopaskal (kPa), pH >7.25, and positive end-expiratory pressure (PEEP) <6 cmH_2_O.

1.c. Hemodynamic stability with respect to use of vasopressors (noradrenaline <0.1 γ) and/or inotropes (dobutamine <5 γ).

1.d. The ability to deliver the work of breathing with respect to negative inspiratory pressure greater than -5 cmH_2_O and adequate tidal volume.

2. The two methods of carrying out the spontaneous breathing trial (SBT) that are used in our daily clinical practice (T-piece system or continuous positive airway pressure of 5 cmH_2_O).

3. Criteria to evaluate the SBT, including:

3.a. Gas exchange remains adequate with respect to pH >7.35, change in carbon dioxide partial pressure (pCO_2_) <1.3 kPa, pO_2 _>7.4 kPa and oxygen saturation >90%.

3.b. Hemodynamic stability is not impaired with respect to heart rate (HR) <130 beats/min, change in HR <20%, systolic blood pressure (BP) 90 to 200 mmHg and change in BP <20%.

3.c. Ventilation pattern remains stable with respect to respiratory rate (RR) <30 breaths/min and change in RR <50%.

3.d. Subjective tolerance of the patient with respect to signs of distress and vasovagal signs.

### Data collection

Within 12 hours after the occurrence of an UE, the researcher filled out the standardized data collection tool (DCT) on the basis of the electronic medical and nursing records. For a control patient, the same DCT was filled out. Additional information was obtained by using a standardized questionnaire for every case and control by interviewing the nurse who witnessed or discovered the UE or who was involved in the care of the control patient. The standardized questionnaires were based on a comprehensive literature review of previous UE studies [1-4,6,16-18,21,22] and on practical insights from the medical ICU staff. Information on complications and reintubations (within 48 hours) following the UE was obtained for each patient. Data extraction and monitoring of follow-up were equal for cases and controls.

Several strategies were established to enhance the implementation of the study. Information sessions were held before the start of the study to educate the ICU nurses and doctors about the study's aim and procedures. Attention posters were clearly posted in all ICU units, and a researcher visited the subunits daily to record the number of intubated patients and to implicitly remind the ICU staff about the study. Moreover, the researcher received a bimonthly report from the ICU incident database. In this database, incidents such as UEs occurring on the ICU were registered. According to this database, no UEs were missed. To select controls, all mechanically ventilated ICU patients were assigned a number, and a random number generator selected four numbers. The four selected patients represent the control patients. Identical information was collected for these control patients.

### Statistical analysis

Continuous and ranked variables were compared using the Student's *t*-test or the Wilcoxon rank-sum test in cases of non-normal distribution and expressed, respectively, as means ± SD or median and interquartile range. Categorical variables were expressed as percentages and analyzed using a χ^2 ^test.

To determine independent risk factors for UE, univariate logistic regression was used. Determinants significantly associated with UE in the univariate analysis (*P <*0.25) and clinically relevant factors were included in the multivariable logistic regression. All statistics were calculated using SPSS software (version 16.0; SPSS Inc., Chicago, IL, USA).

## Results

### Study population and patient characteristics

In the 30-month study period, 4,255 patients were admitted to the ICU. Of this total, 3,476 patients (82%) required one or more mechanical ventilation periods, resulting in 17,398 ventilation days. Within the study period, 74 UEs occurred in 69 patients. Five patients experienced an UE twice. A total of 296 controls were included.

Demographic and clinical characteristics are reported in Table 1. Of all cases, the majority were male (77%). The median age of the cases was 61 years (range, 47 to 75 years). UEs occurred more frequently during night shifts (38%) than during day or evening shifts. Cases and controls did not differ significantly with respect to age and diagnosis category or type of admittance.

**Table 1:** Demographic and clinical characteristics of patients and controls^a^

	UE Cases	Controls	
Variables	(*n *= 74)	(*n *= 296)	*P *value
Mean age, yr (± SD)	60.6 (± 14)	61.2 (± 16)	0.22
Males, *n *(%)	57 (77%)	188 (64%)	0.03
APACHE II score (± SD)	16.4 (± 8)	18.5 (± 8)	0.36
Type of admittance^b^, *n *(%)			
Medical	34 (46%)	135 (46%)	0.82
Planned surgery	30 (43%)	128 (43%)	0.79
Urgent surgery	10 (14%)	33 (11%)	0.65
Cardiovascular	39 (53%)	124 (42%)	0.85
Diagnostic category, *n *(%)			
Respiratory	17 (23%)	74 (25%)	0.33
Sepsis	2 (3%)	12 (4%)	0.41
Neurological	9 (12%)	39 (13%)	0.41
Gastrointestinal	5 (7%)	33 (11%)	0.15
Vascular	1 (1%)	6 (2%)	0.45
Metabolism	1 (1%)	1 (0.3%)	0.56
Hematological	-	3 (1%)	0.99
Renal	-	3 (1%)	0.99

^a^UE, unplanned extubation; APACHE II, Acute Physiology and Chronic Health Evaluation; SD, standard deviation; ^b^Type of admittance: *Medical*, no surgery in the week before intensive care unit admission; *Planned surgery*, planned surgery; *Urgent surgery*, immediate surgery where resuscitation, stabilization and physiological optimization simultaneously took place with surgery.

### Incidence of unplanned extubations

Of 3,476 patients requiring mechanical ventilation, UEs occurred in 69 patients. This translates to an UE incidence of 2.0% for mechanically ventilated patients. The incidence rate of UEs was 0.004 per ventilation day.

### Determinants of unplanned extubations

Determinants that were associated with an UE in the univariate analysis are provided in Table 2. Male sex, higher body mass index (BMI), ICU subunit B with preferential surgical patients, an elevated serum sodium level at time of UE, low Ramsay Sedation Scale score (anxious/agitated and awake/cooperative) and use of haloperidol and methadone at the time of UE were associated with an increased risk of UE.

**Table 2:** Univariate and multivariable analysis: determinants associated with unplanned extubation^a^

	Univariate analysis		Multivariable analysis	
		
Variables	OR (95% CI)	*P *value	OR (95% CI)	*P *value
Sex^b^	1.9 (1.07-3.48)	0.03	1.8 (0.84-3.89)	0.13
Age (yr)	0.9 (0.98-1.01)	0.76	1.0 (0.97-1.03)	0.98
BMI (kg/m^2^)	1.1 (1.00-1.10)	0.04	1.0 (0.97-1.11)	0.26
Subunit ICU^c^		0.02		
ICU subunit A	1.2 (0.62-2.41)	0.60	1.0 (0.38-2.42)	0.94
ICU subunit B	2.2 (1.23-4.02)	0.01	2.6 (1.06-6.53)	0.04
Length of stay at time of UE (days)^d^	0.9 (0.97-1.00)	0.07	1.0 (0.93-0.99)	0.01
Sodium (mmol/L) at time of UE	1.0 (1.00-1.09)	0.05	1.0 (0.97-1.11)	0.26
Ramsay Sedation Scale score at time of UE		<0.01		<0.01
1 Anxious/agitated	41.4 (4.84-354.05)	<0.01	30.6 (3.18-294.20)	<0.01
2 Awake/cooperative	15.2 (1.96-117.89)	<0.01	25.5 (2.99-216.96)	<0.01
3 Responds to commands only	6.4 (0.77-53.29)	0.09	7.0 (0.78-63.01)	0.08
4 Brisk response to loud noise	3.0 (0.29-31.01)	0.34	1.4 (0.12-15.97)	0.79
5 Sluggish response to loud noise	2.8 (0.29-26.59)	0.37	1.8 (0.17-18.42)	0.62
6 No response (reference)	1.0 (reference)	-	1.0 (reference)	-
Clonidine use at time of UE	2.3 (0.97-5.33)	0.06	2.3 (0.67-7.56)	0.19
Haloperidol use at time of UE	2.1 (1.24-3.51)	0.01	1.6 (0.66-3.72)	0.31
Methadone use at time of UE	2.0 (1.07-3.65)	0.03	0.9 (0.39-2.46)	0.97
Midazolam use at time of UE	1.4 (0.83-2.31)	0.21	2.3 (1.01-5.18)	0.05
Other benzodiazepine use at time of UE (diazepam, lorazepam, oxazepam, temazepam)	1.5 (0.85-2.55)	0.16	1.1 (0.48-2.69)	0.77

^a^OR, odds ratio; 95% CI, 95% confidence interval; BMI, body mass index; ICU, intensive care unit; UE, unplanned extubation; ^b^Reference category is female; ^c^Reference is ICU subunit C; ^d^Index time, sampling time for controls and time of UE for patients.

In the multivariable analysis, the following variables were associated with UE: ICU subunit, with an increased risk of UE in subunit B (OR, 2.6; 95% confidence interval (95% CI), 1.06 to 6.53), length of stay (index time) in the ICU with an increased risk for patients with a shorter length of stay (OR 0.9; 95% CI, 0.93 to 0.99), first (anxious/agitated) and second (awake/cooperative) categories of the Ramsay Sedation Scale (OR, 30.6; 95% CI, 3.18 to 294.20; and OR, 25.5; 95% CI, 2.99 to 216.96, respectively) and midazolam use at the time of UE (OR, 2.3; 95% CI, 1.01 to 5.18).

### Follow-up after unplanned extubations

Patients with an UE had significantly lower hospital mortality than patients without an UE (19% versus 32%; *P *= 0.028). The difference persisted after correction for the severity of disease (Acute Physiology and Chronic Health Evaluation II), age and type of admission (OR, 0.5; 95% CI, 0.28 to 1.00). Furthermore, patients with an UE had a shorter total intubation time (*P *= 0.074) and lower ICU mortality (*P *= 0.096), although these associations did not meet the criteria for statistical significance (Table 3).

**Table 3:** Clinical outcomes comparing patients who underwent UE with mechanically ventilated controls^a^

Outcome	Patients (*n *= 74)	Controls (*n *= 296)	Mean difference (95% CI)	*P *value
Mean ICU-LOS at index time^b^, days	10	14	4 (0.68-8.34)	0.021
Mean ICU-LOS after UE, days	14	16	2 (3.20-7.36)	0.436
Mean length of total intubation time, days	23	29	6 (6.44-13.75)	0.074
Mean LOS ICU, days	24	30	6 (1.15-13.78)	0.097
Mean LOS hospital, days	43	48	5 (6.19-14.97)	0.413
ICU mortality, *n *(%)	13 (18)	80 (27)	-	0.096
Hospital mortality, *n *(%)	14 (19)	95 (32)	-	0.028

^a^UE, unplanned extubation; ICU, intensive care unit; LOS, length of stay; ^b^Index time, sampling time for controls and time of UE for cases.

Forty-seven percent (35 of 74) of all UE patients did require reintubation, and 53% did not need reintubation. Of the patients who had to be reintubated, all reintubations occurred within 12 hours of the UE (89% within 1 hour and 11% between 1 and 12 hours). Moreover, 66% of patients had to be reintubated between 0 and 29 minutes and 23% of patients had to be reintubated between 30 and 59 minutes. Table 4 compares patients with an UE who did not need reintubation with patients who did need reintubation. Patients without reintubation had a significantly shorter length of stay in the ICU and in the hospital (10 days versus 40 days and 28 days versus 61 days, respectively), shorter duration of total intubation time and lower ICU and hospital mortality. Thus, the outcome after UE seemed to depend on the need for reintubation.

**Table 4:** Clinical outcomes comparing patients who underwent UE with need for reintubation with patients who underwent UE without reintubation after UE^a^

Outcome cases	UE with reintubation (*n *= 35)	UE without reintubation (*n *= 39)	Mean difference (95% CI)	*P *value
Mean LOS ICU, index time^b ^(days)	13	7	6 (12.29-0.23)	0.059
Mean LOS ICU, after UE (days)	26	3	23 (31.68-15.27)	<0.001
Mean length of total intubation (days)	38	9	29 (40.73-18.70)	<0.001
Mean LOS ICU (days)	40	10	30 (41.43-18.18)	<0.001
Mean LOS hospital (days)	61	28	33 (51.86-15.14)	<0.001
Mortality ICU, *n *(%)	13 (37)	0 (0)	-	<0.001
Mortality hospital, *n *(%)	13 (37)	1 (3)	-	<0.001

^a^Following unplanned extubation (UE) (*n *= 74), patients without reintubation had significantly better outcomes than reintubated patients; 95% CI, 95% confidence interval; LOS, length of stay; ICU, intensive care unit; ^b^Index time, sampling time for controls and time of UE for cases.

Risk factors for reintubation after an UE were the level of PEEP (*P *= 0.05) and respiratory frequency before UE (*P *= 0.05). The mode of mechanical ventilation was not significantly associated with reintubation after an UE (*P *= 0.428). Furthermore, patients with pulmonary comorbidity had an increased risk of needing reintubation after an UE (*P *= 0.024). During the ICU stay, delirium and respiratory problems were other factors associated with the need for reintubation after an UE (*P *= 0.021 and *P *= 0.027, respectively).

## Discussion

This study has shown that UE occurred in 2% of all mechanically ventilated patients. Being awake or being agitated (Ramsay Sedation Scale scores 1 and 2, respectively), use of midazolam and being admitted to a specific ICU subunit were associated with an increased risk for UE. Analysis demonstrated that patients with an UE without subsequent need for reintubation had lower ICU and hospital mortality than mechanically ventilated controls and UE patients in need of reintubation.

In the present study, we used a case-control design, which was also used in some other studies [1,5,15,17,18]. The case-control design enables researchers to study the relationship of multiple factors for one outcome and is especially appropriate for studying infrequent outcomes, such as UE. However, the selection of controls is crucial for a case-control study. The control group should be a random sample of all patients who were at risk of experiencing the studied outcome. In the present study, the control group was sampled from all other mechanically ventilated patients admitted to the ICU at the time of an UE. These patients are in principle at risk for an UE and represent the distributions of risk factors that will be compared to the distribution of risk factors in the cases, with respect to the level of sedation. Both cases and controls had a Ramsay Sedation Scale score vary from 1 to 6, pointing toward the appropriateness of the control group. We applied density sampling, and control patients were matched on time. Consequently, it was possible that a patient served as a control twice. Nevertheless, at both time points, the control patients were truly representative of the population from which the case arose and were comparable to the case of that time point.

The reported incidences of UEs differ largely, ranging from 0.3% to 14% [7,10,13,15,18]. These incidence levels are difficult to compare because of differences in the calculation method used for data collection in the various studies. Furthermore, the incidence variation can be partially explained by the heterogeneity of the studied ICU population [14]. The incidence of UEs in our ICU was relatively low (2.1% for mechanically ventilated patients and 0.4% per ventilation day). This can partly be explained by the high nurse-to-patient ratio in our hospital. It is unlikely that underreporting is responsible for the low incidence, since in our institution parallel incident reporting systems at the ICU are used to minimize this effect. Furthermore, extensive attention was given to the implementation and execution of the study.

Male sex and subunit ICU were risk factors for UE. Remarkably, not only agitation and restlessness (Ramsay Sedation Scale score 1) predisposed patients to UE, but also normal consciousness (Ramsay Sedation Scale score 2) was highly associated with UE. Our findings are consistent with those reported by other authors [1,11,13,17,18,23,24]. The observed agitation and restlessness could well be the clinical manifestations of delirium. We were capable of investigating all medication use (narcotics and analgesics) at the time of UE by means of the electronic medical records. The proportion of patients who received sedatives and narcotic analgesics was similar between the two groups. In the univariate model, we found that the medication administered to decrease agitation and delirium actually increased the risk of an UE. In the multivariable model, midazolam was associated with an increased risk of UE. A possible explanation is that midazolam is known for its paradoxical reaction [25] and is also associated with delirium in ICU patients [26]. Furthermore, the relationship could be confounded by the facts that agitated patients were more frequently treated with midazolam and we were unable to completely correct for agitation. To get more insight into this process, a future randomized, controlled trial has to be established that could focus on the dose-response relationships and on goal-directed medication use. The ICU subunit with an increased risk for UE distinguishes itself by a somewhat higher admittance rate of postoperative cardiosurgical patients. It is known that this patient subgroup is more likely to be agitated after surgery [27]. Although we corrected for type of patient (medical, surgical or thoracic surgical), this could be the explanation for the subunit effect. Factors that might have been a clarification in terms of subunit culture or care were not systematically collected and therefore were not examined.

Previous studies [1-12], particularly before 2000, showed that UE was associated with a higher risk for prolonged duration of mechanical ventilation, increased ICU stay, increased hospital stay and increased mortality. In the present study, patients with an UE had better outcomes than control patients. This finding is not yet very well established in the literature on UE, although we are not the first investigators to report it. Epstein *et al*. [5], Krinsley and Barone [15] and Bouza *et al*. [14] also found that the outcome after an UE was than that of patients without UEs. A first explanation could be that UE patients are in better clinical condition, more alert, physically stronger and able to extubate themselves. Although it is not obvious from the baseline characteristics (Table 1), we cannot exclude that this explanation is valid. Second, earlier UE could result in shorter duration of mechanical ventilation and ICU length of stay and thus in fewer complications and improved outcomes. The improved outcomes of the UE patients not needing reintubation is in accord with this hypothesis. Only a few other authors [5,14-16] have described outcome differences between patients with UE with a subsequent need for reintubation and patients with UE not needing a reintubation. Krinsley and Barone [15] and Bouza *et al*. [14] were the first to describe better outcomes for patients who experienced an UE and did not need reintubation.

Another explanation for the improved outcomes is that patients at our ICU are systematically intubated longer than they need to be. We do not know at what time patients with UEs would have had their planned extubations, but we hypothesized that the extubation success rate of the overall ICU population might provide additional insight. We have calculated our historic extubation success rate. In accordance with other reports in the literature, we defined the extubation success rate as the proportion of patients in whom it was unnecessary to reinstitute ventilatory support within 48 hours after planned extubation [28]. Over the study period (1 January 2005 to 1 June 2008), we found that of 4,710 patients, 931 needed reintubation within 48 hours after planned extubation, resulting in an extubation failure rate of 19.8% and an extubation success rate of 80.2%. These data could suggest that a proportion of our patients were indeed intubated longer than they needed to be despite our weaning protocol, therefore putting them at increased risk of UE. Maybe because it is not explicitly stated in our protocol how often patients should undergo a SBT, the SBT is not applied as frequently as it should be to select patients who are eligible for extubation. Another hypothesis is that the medical and nursing staff are still reluctant to perform extubation despite the use of a weaning protocol. Awareness and education with respect to the weaning protocol and more explicit discussion of the frequency of SBTs could decrease this reluctance to perform extubation and thereby reduce the length of mechanical ventilation time and maybe minimize the UE rate [1,2,18,29,30].

With respect to the UE patients who needed reintubation, it can be stated that in our institute, a protocol for intubation and the start of mechanical ventilation is applied with the following intubation criteria: (1) upper airway obstruction, (2) respiratory failure due to exhaustion, (3) impaired or decreased level of consciousness, (4) cardiopulmonary arrest and (5) need for sedation for diagnostic or therapeutic procedures. The fact that 90% of the reintubated UE patients needed reintubation within 1 hour is in agreement with the risk factors for reintubation (high PEEP, respiratory frequency for UE, delirium during ICU stay and respiratory problems during ICU stay).

Some studies [1,13,18] have developed a clinical risk stratification tool to identify patients at risk for UE. These tools are based mainly on significant sedation and the consciousness level of the patient. Our study observed additional risk factors. If patients are identified as having a high risk for UE, temporarily intensified surveillance may be needed and extubation should be performed as soon as possible. ICU staff could take additional preventive measures (for example, preventing agitation, adjusting clear fixation policy, enforcement of 24-hour bedside supervision). These new policies will be an important area for further research investigations.

## Conclusions

ICU patients who experienced an UE did not have increased mortality. Moreover, following an UE, patients not needing reintubation had significantly better outcomes than reintubated patients. Male sex, subunit ICU, agitated or awake consciousness and use of midazolam at the time of UE were identified as risk factors for an UE.

## Key messages

• We have introduced additional risk factors for unplanted extubation. Use of midazolam and being admitted to a specific ICU subunit were associated with an increased risk for unplanted extubation (UE).

• Analysis demonstrated that patients with an UE without subsequent need for reintubation had lower ICU and hospital mortality than mechanically ventilated controls and UE patients in need of reintubation.

• Medical and nursing staff may be reluctant to administer extubation.

• Introduction of weaning and/or extubation protocols and daily evaluation of the need for mechanical ventilation could reduce the length of mechanical ventilation time and minimize the UE rate.

## Abbreviations

APACHE: Acute Physiology and Chronic Health Evaluation; BMI: body mass index; BP: blood pressure; DCT: data collection tool; FiO_2_: fraction of inspired oxygen; HR: heart rate; ICU: intensive care unit; LOS: length of stay; pCO_2_: carbon dioxide partial pressure; pO_2_: oxygen partial pressure; PEEP: positive end-expiratory pressure; RR: respiratory rate; SBT: Spontaneous Breathing Trial; UE: unplanted extubation; γ: gamma (=microgram/kilogram/minute).

## Competing interests

The authors declare that they have no competing interests.

## Authors' contributions

RG participated in the design and coordination of the study, carried out the data collection, performed the statistical analysis and drafted the manuscript. OD participated in the design of the study and sequence alignment of the manuscript. IH carried out part of the data collection. EJ participated in the sequence alignment of the manuscript. MA conceived the study, participated in the design of the study, performed the statistical analysis and participated in the sequence alignment of the manuscript. All authors read and approved the final manuscript.
